# Reviewing bryophyte-microorganism association: insights into environmental optimization

**DOI:** 10.3389/fmicb.2024.1407391

**Published:** 2024-06-14

**Authors:** Bhagyashri V. Dangar, Pratikkumar Chavada, P. J. Bhatt, Rajesh Raviya

**Affiliations:** Department of Life Sciences, Bhakta Kavi Narsinh Mehta University, Junagadh, Gujarat, India

**Keywords:** bryophyte, microorganism, symbiotic association, bacterial and fungal endophytes, nitrogen dynamics

## Abstract

Bryophytes, the second-largest group of plants, play a crucial role as early colonizers of land and are a prolific source of naturally occurring substances with significant economic potential. Microorganisms, particularly bacteria, cyanobacteria, fungi form intricate associations with plants, notably bryophytes, contributing to the ecological functioning of terrestrial ecosystems and sometimes it gives negative impact also. This review elucidates the pivotal role of endophytic bacteria in promoting plant growth, facilitating nutrient cycling, and enhancing environmental health. It comprehensively explores the diversity and ecological significance of fungal and bacterial endophytes across various ecosystems. Furthermore, it highlights the moss nitrogen dynamics observed in select moss species. Throughout the review, emphasis is placed on the symbiotic interdependence between bryophytes and microorganisms, offering foundational insights for future research endeavors. By shedding light on the intricate bryophyte-microorganism associations, this study advances our understanding of the complex interplay between plants, microbes, and their environment, paving the way for further research and applications in environmental and biotechnological realms.

## 1 Introduction

Bryophytes, belonging to the Bryophyta division, boast an incredible diversity, with over 23,000 species distributed worldwide. Classified into three main groups - mosses (class Bryopsida), liverworts (class Hepaticopsida), and hornworts (class Anthocerotopsida), these plants thrive in a wide range of environments, making them crucial components of ecosystems (Bahuguna et al., 2013). They are considered the second most diverse group of plants after flowering plants, and are believed to be among the oldest terrestrial plants (Clarke et al., 2011). Being early colonizers of land, bryophytes faced numerous challenges, including pathogen attacks and insect predation, due to their exposure to adverse environmental conditions (Whitehead et al., 2018a). Symbiotic relationships between bryophytes and microorganisms, including fungi, bacteria, and algae, are vital for the ecological functions and survival strategies of these plants. Fungi aid in nutrient absorption, especially in nutrient-poor soils, while nitrogen-fixing bacteria enable bryophytes to thrive in nitrogen-deficient environments. Algae, particularly cyanobacteria, contribute to photosynthesis, enhancing the production of organic compounds crucial for bryophyte growth. These symbiotic associations increase resilience to environmental stresses, enabling bryophytes to occupy diverse habitats worldwide, from rocky terrains to polar regions, and play essential roles in ecosystem functioning (Adams and Duggan, 2008; Rimington et al., 2018; Glime, 2019; Poveda, 2020).

Microorganisms like algae (including cyanobacteria), bacteria, and some macro fungi are associated with bryophyte including all three classes i.e., liverwort, moss and hornwort. Bryophytes may potentially be parasitized by a wide variety of fungus, lichens, and microbes (During and Tooren, 1990). Since the majority of mosses are ectohydric, the gametophytes can absorb water and dissolved minerals onto their surfaces. The moss leaf surface is comparable to the rhizosphere in this manner. This could be among the factors responsible for the colonization of microorganisms (Opelt and Berg, 2004). Additionally, the related population of microorganisms varies according to the host's needs; for example, cyanobionts are in variable phases of forming symbiosis in different sections of the thallus, as shown by varying heterocyst frequency and enzymatic activity, this leads to metabolically diverse cyanobiont populations (Rai et al., 1989). Unlike flowering plants, liverworts are more often linked to ancient lineages of arbuscular mycorrhizal fungus, while being some of the closest extant relatives of the first land plants (Rimington et al., 2018). An essential component of the nitrogen economy of terrestrial arctic habitats is the biological fixation of atmospheric nitrogen by cyanobacteria associated with mosses (Solheim et al., 2004). The interactions that bryophytes have with a wide range of species are diverse and can range from obligatory symbioses to sporadic epiphytism. Over time, a qualitative understanding of the many kinds of interactions, the species involved, and certain structural traits of the connections have been described (During and Tooren, 1990).

The greatest source of naturally occurring substances with potential economic value is microorganisms. In addition to being a source of polyunsaturated fatty acids, endophytes, such as fungi and bacteria that intracellularly colonize plant tissues, are known to be a rich source of new compounds, including anticancer drugs, antibiotics, antivirals, antioxidants, and immunomodulatory substances. (Brady and Clardy, 2000; Wrigley, 2000; Strobel and Daisy, 2003; Bérdy, 2005). In plants, endophytes are a widespread world. Over the course of long-term coevolution, they have developed a mutually beneficial connection with host plants. A complex microecosystem is made up of the diverse range of microbial species that make up the endophytic community. Numerous studies have demonstrated that endophytes directly produce bioactive compounds that protect their host plants from harmful microorganisms and herbivores, increasing the fitness of the host plants (Stelmasiewicz et al., 2023). It's possible that endophytic fungi are abundant sources of naturally occurring bioactive substances that have use in the pharmacological, medical, and agricultural sectors (Govindan and Venkatesan, 2022). The endophytic bacteria support the growth of host plants by fortifying their resilience to both biotic and abiotic stressors (Stelmasiewicz et al., 2023). Certain chemicals found in bryophytes have antibacterial and antibiotic qualities (Bahuguna et al., 2013). So, the use of bryophytes as a source of microorganisms to enhance crops and/or forest species is now understudied (Poveda, 2024).

This review sheds light on the significant yet often overlooked associations between bryophytes and microorganisms. Exploring the bryophyte-microbe association would help in better understanding of the terrestrial ecosystems. Future research can prioritize this area by employing molecular techniques, investigating environmental influences, and integrating computational modeling. Such efforts deepen our understanding of ecological networks and symbiotic interactions, offering avenues for environmental sustainability and biotechnological innovation.

**Figure 1:**
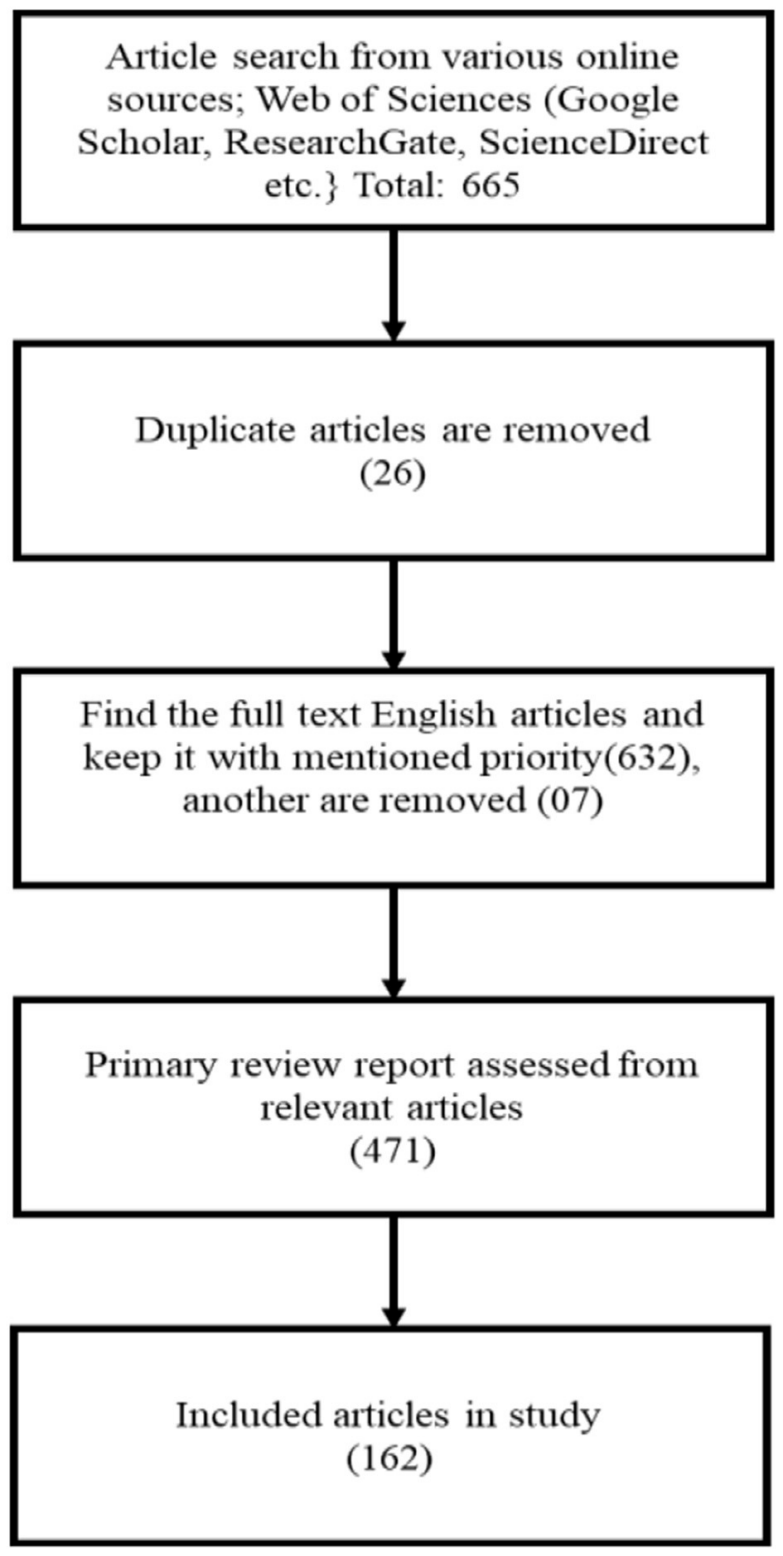
Flow diagram of article analysis [adapted from Chen and Nelson (2022)].

The flow diagram (Figure 1) of article analysis illustrates the systematic process of evaluating and synthesizing information from scholarly articles. It typically begins with the identification of relevant articles through database searches, followed by screening for eligibility based on predefined criteria. The next step involves assessing the quality and relevance of the selected articles, often through methods such as critical appraisal or risk of bias assessment. Subsequently, data extraction is performed to gather key information from each article, such as study design, participant characteristics, interventions, outcomes, and results.

## 2 Diversity of bryophyte-microbe association

The associations between microorganisms and plants, particularly bryophytes, encompass a diverse array of interactions crucial for the ecological functioning of terrestrial ecosystems. These associations can be categorized into several types: (i) Endophytes reside within healthy plant tissues, potentially exerting various impacts on the host, from beneficial to pathogenic; (ii) Epiphytes, on the other hand, inhabit the surface of plant hosts, forming a microbiota that contributes to nutrient cycling and defense mechanisms; (iii) Mycorrhizal relationships represent mutualistic partnerships between fungi and plant roots or other structures, facilitating nutrient exchange and enhancing plant growth, with various types such as arbuscular, ecto, and ericoid mycorrhizae; (iv) Mycoheterotrophy involves non-photosynthetic plants acquiring carbon resources through fungal partners, showcasing the intricate web of interactions shaping bryophyte ecosystems and highlighting their importance in nutrient cycling and ecosystem resilience (Chen and Nelson, 2022).

Table 1 summarizes the associations between bryophytes (mosses, liverworts, hornworts) and microorganisms (bacteria, fungi, virus, algae). These relationships are crucial for the ecology and physiology of both groups. The table details specific bryophyte species and their associated microorganisms, along with the types of symbiotic relationships formed. References provide further insights into the supporting research. This compilation is a valuable resource for understanding the interactions between microorganisms and bryophytes.

**Table 1:** Association of microorganisms with bryophyte species.

**Group of microorganisms**	**Microorganism species**	**Bryophyte species**	**Type of interaction**	**Classes of bryophytes and effect (if any)**	**References**
**Bacteria**	*Pseudomonas taiwanensis* strain SJPS KUD54	*Anoectangium clarum*	Indirect	Moss	Alam et al., 2019
	*Aeromonas veronii* strain Philippines-An11				
	*Pseudomonas fluorescens* strain D19	*Hyophila involuta*			
	Uncultured α-proteobacterium clone RLBp5566				
	*Routella terrigena* strain 35CL	*Atrichum undulatum*			
	*Halobascillus* sp. HPB32				
	*Actinomadura physcomitrii* sp. nov	*Physcomitrium sphaericum*			Zhuang et al., 2020
	*Burkholderia* sp., *Rahnella aquatilis, Paenibacillus polymyxa, Pseudomonas tolaasii*, and *Streptomyces purpurascens*	*Sphagnum magellanicum* and *Sphagnum fallax*			Opelt et al., 2007b
	Gammaproteobacteria: *Acinetobacter* sp., *Leclercia* sp., *Aeromonas* sp.	*Grimmia montana*			Liu et al., 2014
	Alphaproteobacteria: *Rhizobium* sp., *Brevundimonas* sp., *Methylobacterium* sp.				
	Betaproteobacteria: *Bordetella* sp., *Comamonas* sp., *Methylophilus* sp.				
	Firmicutes: *Planococcus* sp., *Planomicrobium* sp., *Bacillus* sp.				
	*Agrobacterium tumefaciens*	*Marchantia polymorpha*	Direct	Liverwort	Ishizaki et al., 2008 Sugano et al., 2014 Ishizaki et al., 2015 Iwakawa et al., 2021
	*Bacillus subtilis*		Indirect		Yayintaş et al., 2019; Ivković et al., 2021
	*Clavibacter michiganensis*				Ivković et al., 2021
	*Escherichia coli*				Mewari and Kumar, 2008
	*Haemophylus influenzae*				Kámory et al., 1995
	*Listeria monocytogenes*		Indirect		Ivković et al., 2021
	*Methylobacterium* sp.		Direct		Kutschera et al., 2007
	*Methylobacterium marchantiae*				Schauer and Kutschera, 2011
	*Neisseria meningitidis*		Indirect		Kámory et al., 1995; Gahtori and Chaturvedi, 2011
	*Pasteurella multocida*				
	*Proteus mirabilis*				Mewari and Kumar, 2008
	*Pseudomonas aeruginosa*				Kámory et al., 1995
	*Pseudomonas syringae* pv. Tomato		Direct		Gimenez-Ibanez et al., 2019; Matsumoto et al., 2022
	*Staphylococcus aureus*		Indirect		Kámory et al., 1995; Mewari and Kumar, 2008; Ivković et al., 2021
	*Streptococcus pyrogenes*				Kámory et al., 1995
	*Xanthomonas oryzae* pv. *Oryzae*				Gahtori and Chaturvedi, 2011
**Cyanobacteria**	*Nostoc* sp.	*Anthoceros fusiformis*		Hornwort	Costa et al., 2001
	*Nostoc* sp.	*Blasia pusilla*		Liverwort	
	*Nostoc* sp.	*Blasia pusilla, Pleurozium schreberi* and *Hylocomium splendens*		Liverwort and Moss	Warshan et al., 2018
**Fungi**	*Alternaria solani*	*Marchantia polymorpha*	Indirect	Liverwort	Mewari and Kumar, 2011
	*Aspergillus fumigatus*				Sabovljević et al., 2011
	*Aspergillus versicolor*				
	*Bicogniauxia mediterranea*		Direct		Nelson et al., 2018
	*Colletotrichum truncatum*				
	*Daldinia loculata*				
	*Hypoxylon* sp.				
	*Microsphaeropsis arundinis*				
	*Nemania* sp.				
	*Nemania serpens*				
	*Bjerkandera adusta*				Matsui et al., 2020
	*Fusarium oxysporum*		Indirect		Mewari and Kumar, 2011
	*Fusarium oxysporum* f. sp. *lini*				Gahtori and Chaturvedi, 2011
	*F. oxysporum* f. sp. *lycopersici*		Direct		Redkar et al., 2022
	*Penicillium funiculosum*		Indirect		Sabovljević et al., 2011
	*Rhizoctonia solani*				Sabovljević et al., 2011; Poveda et al., 2023
	*Rhizophagus fasciculatus*		Direct		Poveda, 2020
	*Octospora neerlandica* Benkert & Brouwer	*Tortula ruralis* s.str.	Indirect	Develop the infection cushions on rhizoids	Benkert and Brouwer, 2004
		*T. ruraliformis* or *T. virescens*			
	*Octospora fissidentis* Benkert & Brouwer, spec. nov.	*Fissidens bryoides*		Develop the infection cushions on rhizoids	
	*Octospora nemoralis* Benkert & Brouwer, spec. nov.	*Fissidens bryoides*		Infection cushions develop on leaf, stem and rhizoids	
	*Sordaria fimicola*	*Polytrichum commune* and *Ptychostomum capillare*	Indirect		Govindan and Venkatesan, 2022
	*Sporormiella intermedia*				
	*Alternaria alternata*				
	*Aspergillus flavus*				
	*Aspergillus niger*				
	*Cladosporium herbarum*				
	*Curvularia lunata*				
	*Fusarium oxysporum*				
	*Nigrospora orzae*				
	*Trichoderma aureoviride*				
	*Cladophialophora minutissima*	*Polytrichum juniperinum, Aulacomnium palustre*, and *Sphagnum fuscum*		Moss	Davey and Currah, 2006
	*Mortierella alpina*	*Schistidium antarctici*			Melo et al., 2014
	*Smardaea* sp.	*Ceratodon purpureus*			Wang et al., 2011
	*Biscogniauxia mediterranea*	*Marchantia polymorpha*		Liverwort	Nelson et al., 2018
	*Colletotrichum truncatum*				
	*Daldinia loculata*				
	*Hypoxylon submonticulosum*				
	*Nemania serpens*				
	*Phoma herbarum*				
	*Toxicocladosporium irritans*				
	*Xylaria cubensis, Xylaria arbuscula*				
**Virus**	Severe acute respiratory syndrome coronavirus 2 (SARSCoV-2)	*Marchantia polymorpha*	Indirect: *M. polymorpha* metabolites against virus in vitro	*M. polymorpha* metabolites (such as pheophorbide A) have antiviral capacity	Jimenez-Aleman et al., 2021

## 3 Bryophyte-bacteria interactions: unveiling nature's tiny collaborators

Various bryophyte species from the same ecosystem have demonstrated dissimilarities in their bacterial community frameworks (Koua et al., 2015). Vitamin B12 is obtained by at least some, if not all, bryophytes from bacteria; its physiological role is unknown, although it does promote growth and development in culture. The oxidative burst observed in bryophytes during rehydration not only potentially defends against bacterial and fungal pathogens but also highlights a complex microbial interaction that may play a crucial role in the survival strategies of these plants under stressful conditions (Glime, 2022). Strains of *Burkholderia* sp. (ubiquitous, obligately aerobic, rod-shaped, Gram-negative, genus of Pseudomonadota (previously Proteobacteria), *Hafnia* sp. (facultatively anaerobic, rod-shaped, Gram-negative genus of Pseudomonadota), *Methanobacterium* sp. (non-motile, anaerobic genus of Archaea), *Methylobacterium* sp. (pink-pigmented, facultatively anaerobic, straight rod-shaped, Gram-negative genus of Pseudomonadota), *Pantoea* sp. (yellow-pigmented, Gram-negative genus of Pseudomonadota), and *Serratia* sp. (facultatively anaerobic, rod-shaped, Gram-negative genus of Pseudomonadota) are among the numerous bacteria associated with bryophytes in Japan (Opelt and Berg, 2004; Bragina et al., 2013; Koua et al., 2015). However, some bacteria, such as *Bacillus* sp. (Bacillota – synonym = Firmicutes), *Pseudomonas putida* (Pseudomonadota), *Serratia* sp., and *Xanthomonas* sp. (Pseudomonadota), are hostile against the bryophytes (Opelt et al., 2007a). Among the bacterial isolates from the mosses *Sphagnum* and *Aulacomnium, Serratia proteamaculans* and *Serratia liquefaciens* are the most potent antagonists (Opelt and Berg, 2004). According to Alcaraz et al. (2018), microbiomes impact the establishment, growth, uptake of nutrients, defense against pathogens, and overall health of plants. The microbiomes of *Marchantia polymorpha* and *Marchantia paleacea* were compared to those on their respective soil substrates and to plants cultivated from gemmae that were gathered from the same *Marchantia* populations. With a strong and directed effort (by bacteria) to reprogram host cells (of bryophytes) in order to permit, promote, and sustain microbial growth, is how bacteria and bryophytes are related. Upon colonization, hosts undertake a series of intricate regulatory processes to either initiate symbioses or strengthen defenses in order to accommodate or sequester the invading germs (Carella and Schornack, 2018). In the Mesic forests of Hawaii, the nitrogen-fixing bacterium genus *Bradyrhizobium* (Pseudomonadota) establishes a symbiotic relationship with the adventitious roots of its host, *Acacia koa*. (Leary et al., 2004) found that these symbioses produce more and larger nodules in canopy-dwelling mosses than when they are coupled with soil-dwelling roots. Specialized bacteria might be needed for *sphagnum* breakdown, and because of this bacterial specialization, the abiotic environmental variables are more significant than in other systems. The phyla Actinomycetota, Planctomycetota, and Pseudomonadota (Alphaproteobacteria) comprise the majority of these bacteria. According to Kulichevskaya et al. (2007) there aren't many Bacteroidota and Bacillota in eutrophic wetlands, which is thought to be the main decomposer; Planctomycetota populations rose as the decomposition approached its end. There are several ways that bacteria can affect the bryophyte substrates they inhabit. They might aid in the decomposition of dying and dead bryophytes. While some bacteria contribute to the decomposition of decaying bryophytes, thereby facilitating nutrient cycling, others may obstruct light crucial for photosynthesis, thus impacting bryophyte health and productivity. Understanding these dual roles is essential for comprehending bryophyte ecosystem dynamics. However, they could also be respiring and producing CO_2_, which would increase the rates of photosynthetic activity. Beyond these more straightforward duties, however, they can provide hormones and other compounds that could impact the bryophyte's development or the community in which they coexist and relationships are developing that are even more fascinating (Glime, 2022).

## 4 Bryophyte-fungi harmony: exploring nature's symbiosis

Symbiotic relationships between myxomycete and bryophytes manifest through various mechanisms, including unintentional encounters facilitated by favorable environmental conditions such as moisture, and facultative associations where myxomycete opportunistically inhabit bryophyte habitats during their plasmodial stages for sustenance (Glime, 2019). Interestingly, elevation studies indicate that bryophytes can competitively suppress myxomycete through shading or outgrowth at higher altitudes, potentially inhibiting necessary microbial resources via bryophyte-produced antibiotics or alterations in pH levels (Glime, 2019). Moreover, the interplay between bryophytes and microorganisms, including bacteria, protozoa, and algae, may augment the suitability of bryophyte substrates for slime mold colonization (Glime, 2019).

In recent years, extensive research has elucidated fungal associations with bryophytes, shedding light on the evolutionary dynamics and ecological implications of these interactions (Pressel et al., 2014). Molecular and cytological investigations have revealed diverse fungal taxa associating with bryophytes, including Glomeromycetes, Ascomycetes, and Basidiomycetes (Liepina, 2012). Notably, Glomeromycetes exhibit widespread distribution in liverworts, possibly undergoing host swapping from vascular plants (Liepina, 2012). Ascomycetes like *Rhizoscyphus ericae* demonstrate a broad host range, inducing rhizoid branching and septation in leafy liverworts (Valdés et al., 2023). Additionally, observations of arbuscular mycorrhizal (AM) fungal structures in epigeous hepatics underscore the varied fungal associations across bryophyte taxa, with mosses and hornworts showing no evidence of AM interactions (Liepina, 2012).

**Figure 2:**
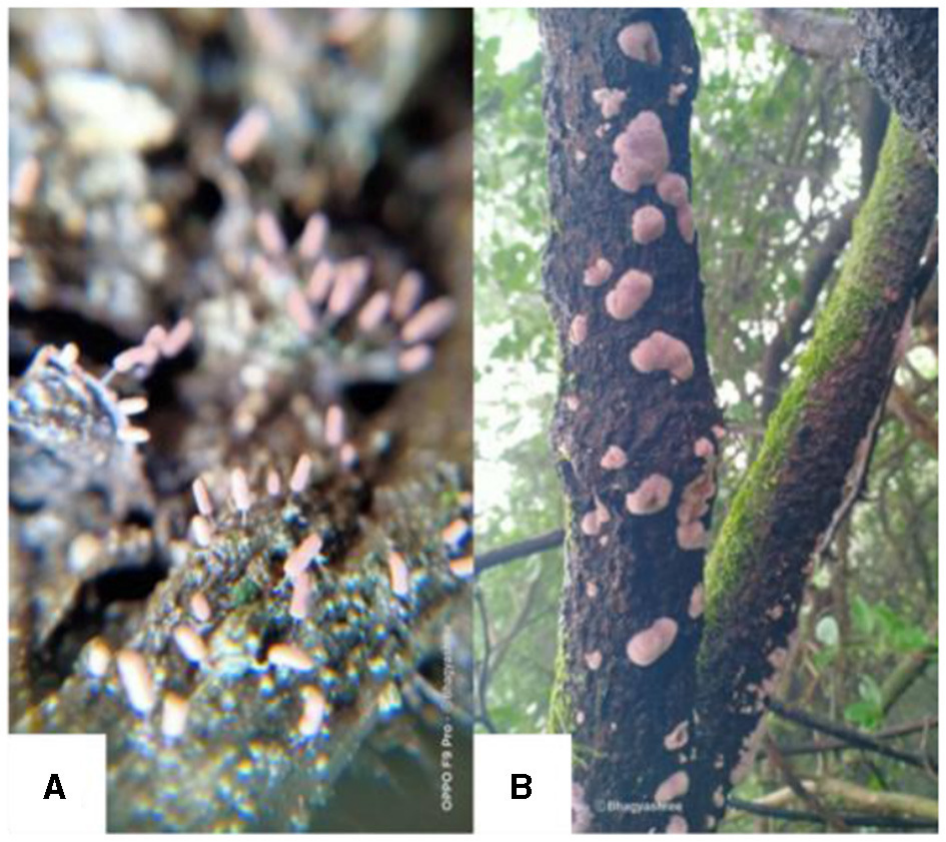
Association of fungi with bryophytes in Junagadh, Gujarat, India. **(A)** Terricolous habitat of bryophyte depicting the association with fungi, **(B)** Corticolous habitat of bryophyte depicting the association with fungi on the bark of *Carissa carandas* L.

Field studies in diverse ecosystems, such as natural reserves in Argentina, have unveiled bryophytes hosting arbuscular mycorrhizal fungi like *Rhizophagus intraradices* and *Dominikia aurea*, highlighting the ecological versatility of bryophytes in varying climatic and environmental conditions (Davey et al., 2013). Fungal communities associated with bryophytes exhibit spatial heterogeneity along elevational gradients, likely influenced by climatic variables and vegetation composition (Davey et al., 2013). Intriguingly, fungi with nematode predatory structures have been observed on deceased bryophytes, hinting at unique ecological roles within bryophyte-associated fungal communities (Davey et al., 2013). Furthermore, bryophytes exhibit intriguing interactions with fungal carpophores, with certain fungi serving as substrates for specific bryophyte species (Davey and Currah, 2006). Bryophilic Pezizales, characterized by distinctive disc-shaped or cupuliform apothecia, demonstrate varying degrees of association with bryophytes, influencing their growth and development through both pathogenic and symbiotic interactions (Norman and Egger, 1999; Davey et al., 2013; Jukić et al., 2020). Overall, these intricate associations underscore the dynamic interplay between bryophytes and fungi, shaping ecological dynamics in diverse terrestrial ecosystems (Figure 2).

## 5 Optimizing environmental nitrogen dynamics through cyanobacterial-bryophyte symbiosis

Mosses, lacking vascular tissue, rely heavily on environmental moisture, making their water retention crucial for survival (Levinsen et al., 2022). The arrangement of moss carpets allows for differential drying rates, with upper sections drying faster, reducing evaporative water loss and maintaining moisture in the lower sections, potentially prolonging Biological Nitrogen Fixation (BNF) activity (Levinsen et al., 2022). Furthermore, mosses exhibit a limited capacity to retain nitrogen, with excess nitrogen leading to significant release, as evidenced by increasing nitrogen concentrations with nitrogen additions in leachates (Rousk and Michelsen, 2016). However, the influence of soil qualities and cyanobacterial colonization introduces variability in moss segment characteristics, impacting their responsiveness to environmental factors like temperature and moisture (Levinsen et al., 2022).

In addition to their dependence on environmental conditions, mosses play a pivotal role in nitrogen dynamics within ecosystems, including nitrogen resorption and release processes (Liu et al., 2020). Despite being non-vascular plants, mosses exhibit efficient nitrogen resorption similar to vascular plants, contributing significantly to nitrogen cycling (Liu et al., 2020). A study on two major forest floor mosses, *Actinothuidium hookeri* and *Hylocomium splendens*, demonstrated their high nitrogen resorption efficiency, making understory mosses nitrogen sinks; however, changing temperature and precipitation patterns could potentially shift their role to nitrogen sources (Liu et al., 2020). This underscores the importance of understanding moss functionality in ecosystem nitrogen dynamics, given the anticipated environmental changes.

Furthermore, moss-cyanobacteria associations play a crucial role in nitrogen fixation dynamics, especially in boreal biomes where molybdenum (Mo) regulates BNF activity (Rousk et al., 2017). The significance of these associations is underscored by the variability in nitrogen fixation rates observed in different environmental settings, such as rural roads exhibiting greater rates compared to busy highways (Ackermann et al., 2012). However, the molecular processes underlying these symbiotic relationships remain largely unknown, necessitating further research to elucidate their formation and maintenance (Alvarenga et al., 2024). Additionally, future changes in temperature and precipitation patterns are expected to significantly impact nitrogen fixation rates in moss-cyanobacteria partnerships, highlighting the need for comprehensive understanding and management of these ecosystems (Rousk et al., 2014).

Moreover, heavy metal exposure near industrial sites may influence moss physiology and associated nitrogen fixation, suggesting potential implications for ecosystem health and nitrogen cycling in polluted environments (Sjøgren et al., 2023). Thus, a holistic approach to studying moss nitrogen dynamics, encompassing environmental factors, symbiotic relationships, and ecosystem responses, is essential for effective management and conservation efforts.

The cyanobacterial symbionts are frequently filamentous and fix nitrogen in specialized cells called heterocysts, which allows them to supply fixed nitrogen and fixed carbon to the host in the case of non-photosynthetic hosts. Typically, the symbionts are *Nostoc* species, which enter the host through specialized motile filaments called hormogonia. Hormogonia form by chemo-attraction, and the host plant generates chemical signals that direct the hormogonia to the site of entry into the plant tissue. Host signals within the symbiotic cavity promote the establishment of heterocysts and dinitrogen fixation while inhibiting further homosexual creation (Thajuddin et al., 2015). There are only few genera where cyanobacterial interactions with liverworts are observed. In comparison, there currently exist 13 genera that have been described for hornworts (Duff et al., 2007). *Nostoc* stands out as the predominant cyanobacteria forming symbiotic relationships with liverworts and hornworts, such as *Nostoc, Blasia*, and *Cavicularia*, exhibit symbiotic associations with bryophytes Despite the different morphology of the *Nostoc* species in culture, the trichomes of these species are so deeply entwined with the cells of the associated bryophytes that it is impossible to distinguish their exact morphology (Duckett et al., 1977; Chauhan et al., 2014). Bryophytes can harbor cultivable actinobacteria, such as *Micromonospora* and *Streptomyces*, which promote plant growth and have plant growth promoting activities encoded in their genomes (Insuk et al., 2020). The presence of cyanobacteria in bryophytes can lead to morphological changes in both the cyanobacteria and the host plants, such as an increase in cavity surface area and proliferation of multicellular filaments (Meeks, 2018). Bryophytes in temperate forests have been found to be associated with cyanobacteria, which provide a substantial nitrogen input to these ecosystems (Deane-Coe, 2015). Cyanobacteria also play a role in mediating interactions between lichens and bryophytes, forming complex biological interactions in epiphytic communities (Cornejo and Scheidegger, 2016). The symbiosis between bryophytes and cyanobacteria is underpinned by complex molecular interactions, including the production of hormogonia-inducing factors that trigger cyanobacterial mobility, intricate cell signaling pathways that guide colonization, and the precise regulation of cyanobacterial cell division within the host tissues (Bouchard et al., 2020). The presence of *Nostoc* in bryophytes leads to changes in the morphology of the host, including an increase in cavity surface area (Meeks, 2018). The diversity of *Nostoc* strains associated with bryophytes is high, with multiple strains being detected within individual thalli (Wang et al., 2022). The symbiotic relationship between *Nostoc* and bryophytes plays a crucial role in nitrogen fixation, as indicated by stable isotope analysis (Costa et al., 2001). Overall, the association of *Nostoc* with bryophytes involves intricate molecular processes and has implications for the adaptation and ecological success of these plants.

Microorganisms, particularly photosynthetic microbes associated with bryophytes, play a crucial role in enhancing carbon dioxide uptake, significantly contributing to carbon sequestration in diverse ecosystems. This interaction not only aids the bryophytes in adapting to varying environmental conditions but also underscores the broader ecological importance of these symbioses in global carbon cycling (Jassey et al., 2022). The contribution of photosynthetic microbes to bryophyte C uptake depends on factors such as the proportion of photosynthetic protists in the moss microbiomes and the environmental conditions. The presence of photosynthetic protists in the moss microbiomes enhances the C uptake, while factors like low moss water content and light conditions can limit their development and reduce overall photosynthetic microbial C uptake (Deane-Coe, 2015; Cornejo and Scheidegger, 2016; Jassey et al., 2022). These findings suggest that microorganisms associated with bryophytes have the potential to support ecosystem-level net C exchanges with the atmosphere, highlighting their importance in the environmental optimization of bryophyte-associated microorganisms (Klaviòa and Spriòìe, 2015; Marzen and Crutchfield, 2018).

**Figure 3:**
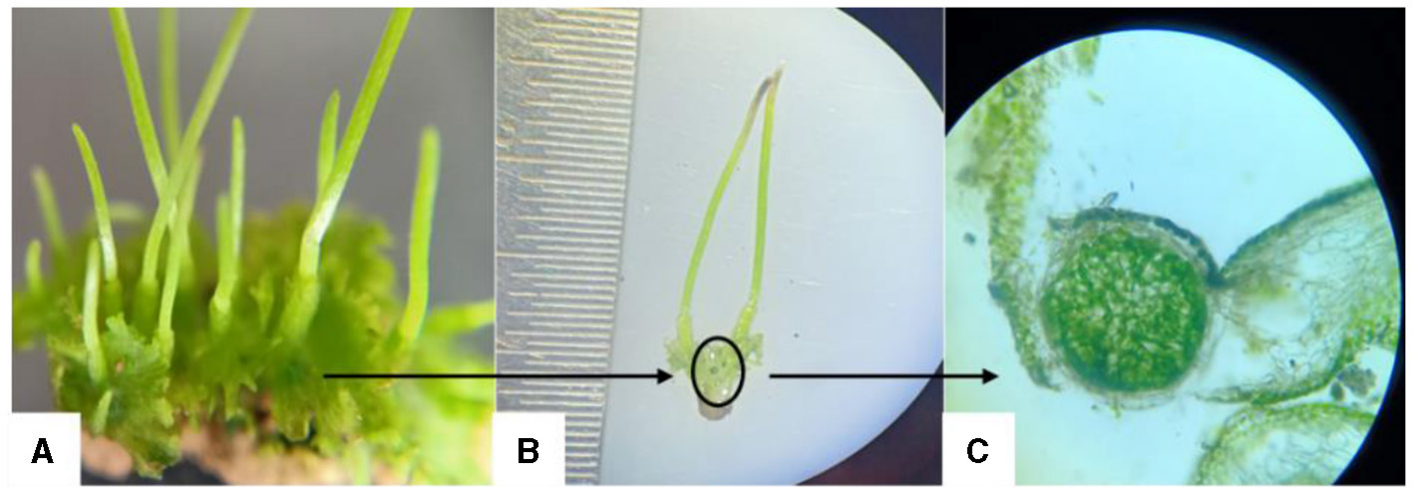
*Anthoceros* sp. (hornwort) thallus inhabiting *Nostoc* colonies; **(A)** Thallus of *Anthoceros* sp., **(B)** circle indicates the dark spot of *Nostoc*, **(C)** T.S. of thallus in light microscope (100X).

Figures 3A–C presents *Anthoceros* sp., a hornwort species, hosting a colony of *Nostoc*, a nitrogen-fixing cyanobacterium. The figure comprises three components: Figure 3A depicts the thallus structure of *Anthoceros* sp., Figure 3B highlights a dark spot indicating the presence of the *Nostoc* colony within the thallus tissue, and Figure 3C shows a transverse section of the thallus under a light microscope, offering insight into the internal organization of the plant tissue and the localization of the *Nostoc* colony. This visual representation elucidates the symbiotic relationship between *Anthoceros* sp. and *Nostoc*, showcasing the integration of the cyanobacterium within the hornwort's structure for mutual benefit.

While symbiotic associations between microorganisms and bryophytes often denote mutualistic relationships, it is imperative to acknowledge that not all interactions result in beneficial outcomes. In fact, instances such as the infection caused by *Atradidymella muscivora* underscore the potential harm that can arise from such associations. This highlights the nuanced nature of bryophyte-microorganism interactions, emphasizing the importance of understanding both beneficial and detrimental outcomes within ecological contexts. for eg. *Atradidymella muscivora* produced floccose, white aerial mycelium on the surface of the gametophytes during the first 10 days of infection, and then it tried to penetrate the protonemata, leaves, and rhizoids. Vegetative hyphae entered the host as early as 5 days after inoculation. They did so by either penetrating the cell walls directly or by producing penetration pegs by the growth of inflated, dome-shaped appressoria, which underwent both terminal and lateral differentiation from the hyphae. The hyphae that penetrated the host cell were often surrounded by a thicker, pigmented deposit that resembled papillae. The papilla-like deposits were classified as simple, bifurcate, or stellate based on how the intruding hypha branched out (Davey et al., 2009).

**Figure 4:**
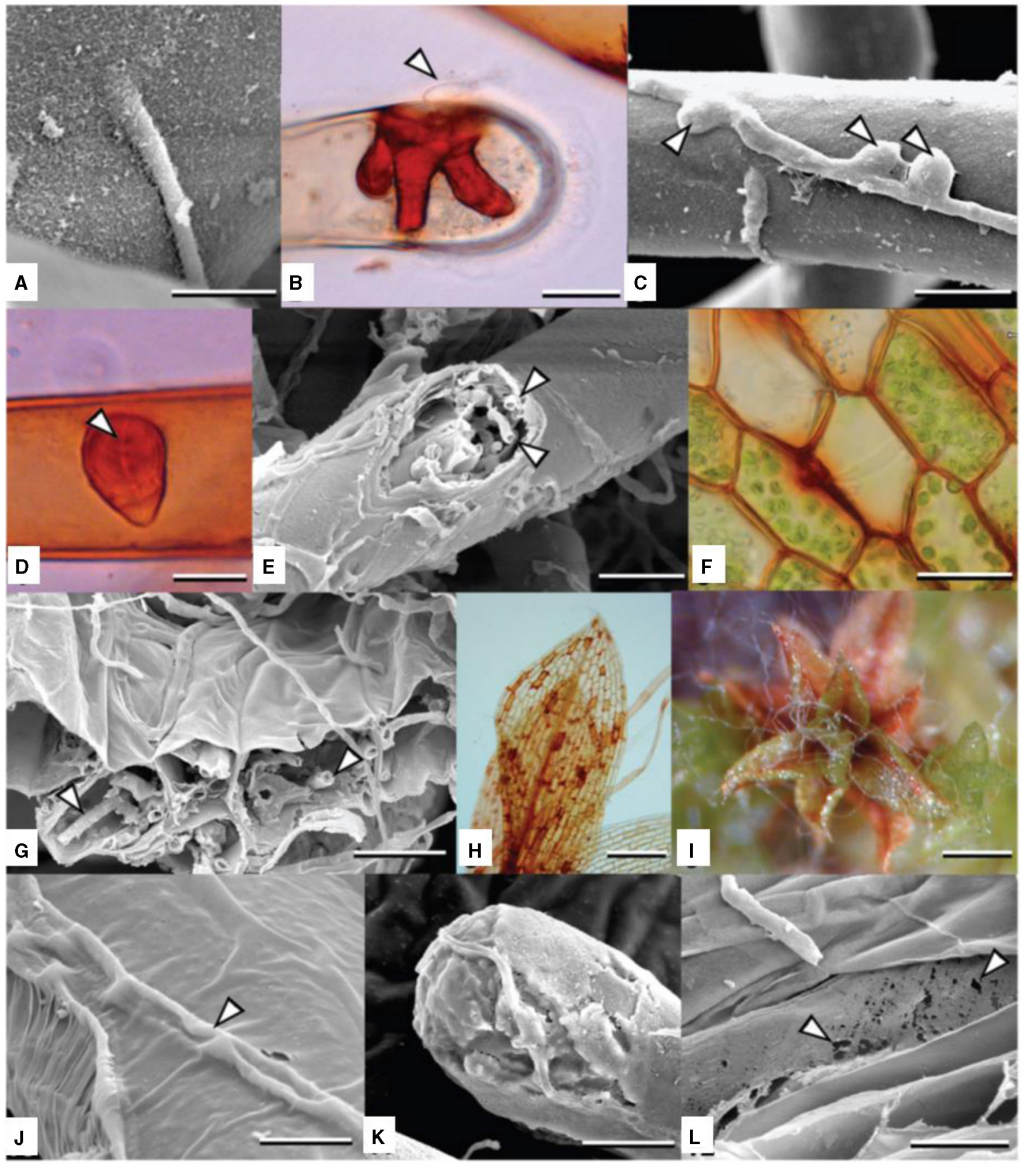
**(A–L)** Colonization and degradation (inducing chlorosis and host death) of *Funaria hygrometrica* by *Atradidymella muscivora*. **(B, D)** lactofuchsin mount; **(F, H)** wet mount. Vegetative hypha directly penetrating host rhizoid. Scale bar = 10 μ m. **(B)** Tip of protonematal filament colonized by *A. muscivora*. The fungus produced an appressorium (white arrowhead) [taken from Davey et al. (2009)].

Figures 4A–L, *A. muscivora* is a generalist pathogen that affects mosses. It exhibits unique adaptations to the bryophyte host in its life cycle, morphology, and patterns of host and microniche exploitation. Because pycnidia grow so quickly, *A. mussidora* could take advantage of wet weather to spread and colonize new hosts, thus extending the disease cycle. The best conditions for conidia to disperse by water are those that arise in a slimy droplet or cirrhus. By guaranteeing that conidia are distributed during times that are favorable for germination, this characteristic may be adaptive by optimizing the likelihood of infection (Davey et al., 2009).

## 6 Conclusions

As a whole, the analysis of bryophyte-microorganism associations offers a sophisticated comprehension of the complex interactions that occurs between bryophytes and a range of microorganisms, such as cyanobacteria, fungus, and bacteria. This review clarifies the significant influence of these relationships on environmental optimization by carefully analyzing the literature and reveals exciting prospects. It is shown that these relationships are critical for the resilience and productivity of ecosystems in processes including organic matter decomposition, nitrogen fixation, and nutrient cycling. Further investigation into bryophyte-microorganism associations is imperative for advancing our comprehension of their intricate ecological roles and interactions. These studies have the potential to unveil profound insights into ecosystem dynamics, biodiversity, and the resilience of natural habitats. Understanding these complex relationships is vital for maintaining ecological equilibrium and addressing the pressing environmental challenges of our time. These applications highlight the transformative possibilities of leveraging these natural symbioses. To harness the full ecological benefits of bryophyte-microorganism associations for promoting sustainable environmental solutions, it is imperative to continue intensive research. Ongoing studies are essential not only to understand the complex dynamics of these interactions but also to develop innovative applications that address pressing environmental challenges. The expanding body of research indicating that fungi and other microbes that are symbiotic or otherwise associated with bryophytes affect their growth and development is not perfectly addressed in this study. It is important to remember that these unnoticed connections may have a significant impact on experimental research.

## Data availability statement

The original contributions presented in the study are included in the article/supplementary material, further inquiries can be directed to the corresponding author.

## Author contributions

BD: Conceptualization, Investigation, Methodology, Validation, Visualization, Writing – original draft, Writing – review & editing. PC: Methodology, Supervision, Validation, Writing – review & editing. PB: Supervision, Writing – review & editing. RR: Conceptualization, Supervision, Validation, Visualization, Writing – original draft, Writing – review & editing.
